# Attempts to Attract Eyesight in E-Commerce May Have Negative Effects

**DOI:** 10.3390/s22228597

**Published:** 2022-11-08

**Authors:** Piotr Sulikowski, Konrad Ryczko, Iwona Bąk, Soojeong Yoo, Tomasz Zdziebko

**Affiliations:** 1 Department of Information Systems Engineering, Faculty of Information Technology and Computer Science, West Pomeranian University of Technology, ul. Zolnierska 49, 71-210 Szczecin, Poland; 2 Department of Applied Mathematics in Economics, Faculty of Economics, West Pomeranian University of Technology, ul. Zolnierska 47, 71-210 Szczecin, Poland; 3 UCL Interaction Centre (UCLIC), University College London, 66-72 Gower Street, London WC1E 6EA, UK; 4 Department of Information Technology in Management, Faculty of Economics, Finance and Management, University of Szczecin, ul. Adama Mickiewicza 64, 71-101 Szczecin, Poland

**Keywords:** highlighting, user attention, e-commerce, eye tracking, website, human-computer interaction

## Abstract

E-commerce shop owners often want to attract user attention to a specific product to enhance the chances of sales, to cross-sell, or up-sell. The way of presenting a recommended item is as important as the recommendation algorithms are to gain that attention. In this study, we examined the following types of highlights: background, shadow, animation, and border, as well as the position of the item in a 5 × 2 grid in a furniture online store, and their relationships with user fixations and user interest. We wanted to verify the effects highlighting had on attracting user attention. Various levels of intensity were considered for each highlight: low, medium, and strong. Methods used for data collection were both implicit and explicit: eye tracking, tracking cart’s contents, and a supplementary survey. Experimental results showed that a low-intensity background highlight should be the first-choice solution to best attract user attention in the presented shopping scenario, resulting in the best fixation times and most users’ selections. However, in the case of the highest-intensity animations, highlighting seemed to have negative effects; despite successful attempts to attract eyesight and a long fixation time, users did not add the highlighted products to cart.

## 1. Introduction

The e-commerce market has been growing rapidly in recent years, with the coronavirus pandemic only accelerating its growth. There is no indication that this trend will reverse. In the United States alone, online sales revenues are expected to grow further by more than 50% by 2025 [1]. It allows sellers to reach many potential customers and often generate a significantly higher income than in brick and mortar stores. Sellers can expand from the local market to a wider area, even the whole world. However, before a potential customer reaches the website, its owner must manage the marketing, site attractiveness, and trustworthiness. Users choose online stores due to their usefulness, while looking for solutions that enable the purchase to be made as quickly as possible [2]. However, traditional stores still have several advantages over online ones. One advantage is the contact between the seller and the customer. The interaction between them is, to some extent, simulated online in the form of recommender systems. This often helps users to reach decisions more efficiently [3]. Why is it so important? The mere entry of the user on the website does not bring any financial benefits. Once the seller has been able to attract a user to their store, they will have a chance to encourage the customer to commit the purchase. Rather, it is only the user’s final decision and payment for the product that can be considered an ultimate success.

We can test user activity on a website based on, for example, mouse cursor movements or scrolling, or by sensing a user’s gaze with eye tracking sensors. To some extent they all reflect the user’s interest [4]. The key goal of recommenders is to show a user a product they prefer, based on their preferences or previous choices. However, this is not always enough—the way in which it is presented to the user also matters [5]. There are other factors that contribute to a positive perception of a store by the customer, such as the level of website interactivity [6], the number of possible options, or the pictures used [7]. Most importantly, recommendation interfaces may include means to additionally highlight some of the recommended items to diversify their content and better attract user attention. However, everything must be completed in moderation. There is a fine line between users’ interest and their annoyance that is easy to cross, which is why attempts to attract user attention might also have opposite effects.

Therefore, the task of website designers is to find a solution that will attract user attention, but will not have a negative impact on their perception, which seems especially applicable to product highlighting. In our study, an online store with furniture was created in a way that the user could view it wholly without the need to scroll. User attention was examined implicitly not to cause additional burden [8], and a survey followed. We tested the following types of product highlights: shadows, colored backgrounds, animation [9,10], and border. Each highlight was tested at 3 levels of intensity to attract user attention and seek optimal solutions for the examined population [11].

It is not difficult to create beautiful interfaces. The trick is to create a website that, in addition to looking good, will allow the user to use it in a simple and pleasant way, saving unnecessary irritation and negative feelings. Although the work on UX is often overlooked, it is as important as the visual layer of the website. A website that is tiring for the user may be discommended. This research aims to assess the plausibility of a hypothesis regarding the possible negative effects of trying to attract the user’s attention with highlights.

## 2. Related Work

Many factors influence the way a user perceives content. This paper examines the effects of different ways of highlighting products. Algorithms that select products for individual users are becoming more effective and there is ongoing work on improving them [12], but their operation was omitted in this study since we focus only on visual highlighting.

Changes in the product grid layout and changes in the positions of recommendations have been already examined by Sulikowski [13], where in a furniture shopping scenario a vertical recommendation layout provided much better results. It was suggested that a horizontal layout can often be associated with unpleasant advertisements such as banners, and the phenomenon of habituation. In addition to the research on optimal location, there have also been discoveries on negative influence of radical visual intensities by Sulikowski and Zdziebko [14].

While designing a shopping site, attention should also be paid to such things as object proportions. Tractinsky et al. [15] tried to explore the advantages of the golden ratio applied to pictures in websites. Due to the mixed results obtained during the experiment, the hypothesis of using the ratio to bring positive results could not be confirmed. It was hypothesized that the golden ratio may have a positive effect on the evaluation of content, but is dependent on the context and other aesthetic forms used.

The timing of showing a highlighted product and website aesthetics may also be of importance. Pappas et al. [16] studied how quickly users will have formed their first impression of a website. The findings indicated that 10 s of viewing time were enough to be able to accurately capture perceptions on visual aesthetics of a website. Pappas et al. [17] also studied how visual aspects of a website relate with users’ gaze patterns, suggesting the need to automatize the process of understanding users’ perceptions to become able to predict user behavior in real time.

One of the better examined means of highlighting was the animation of a product tile. Lee et al. [11] and Li et al. [18] have shown that in the case of banner ads, users pay less attention to the ones that are animated. The participants of the study seemed to know immediately that the object was an advertising banner and tried to avoid focusing on it [19,20]. A thorough study on animation regarding three key animation features (motion, lagging, and looming) and the kind of task performed by the user (browsing and searching) was performed by Hong et al. [9].

The process of assessing the effectiveness of product highlights and their invasiveness in the future may become automatic. There are already attempts to create AI-based methods for reviewing usability [21] and assessing the aesthetics of a page [22,23]—Dou et al. [22] and Khani et al. [23] developed methods using deep learning to automatically compute and quantify webpage aesthetics. It would be interesting to create a similar mechanism to quickly evaluate different methods of product highlighting on an e-commerce website.

All in all, a review of related work showed that highlighting with shadows, colored backgrounds, and borders had not been widely examined, while animation had provided mixed results [9,10], which justifies further studies, including our experiment.

## 3. Experimental Design

In this section we focus on the methodology of the experiment, the course of experimental sessions, the development of the test website, and the sources of data we analyzed.

### 3.1. Experimental Methodology

**Figure 1:**
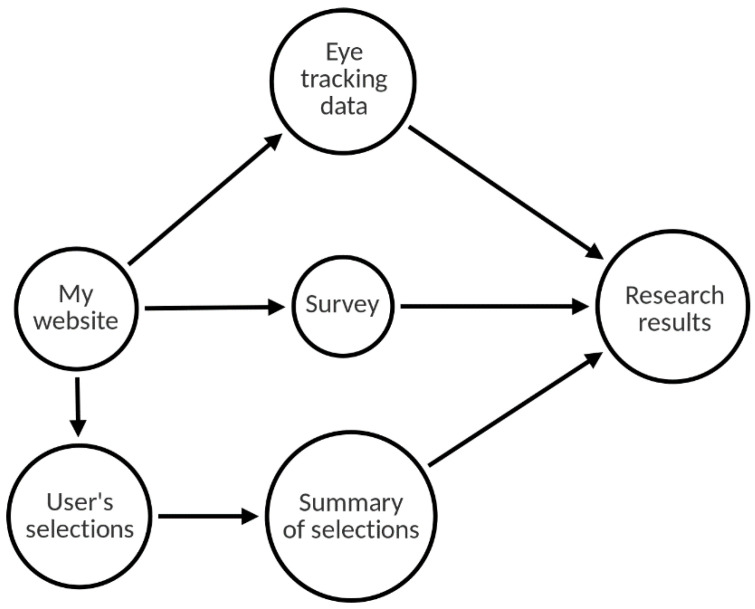
Experimental design summary. Source: own elaboration.

The results in our experiment were obtained based on three forms of data—(1) data on fixation times on individual products obtained from the eye tracker; (2) information about the products selected by users during the study; and (3) a survey carried out after each gaze tracking session. A summary of the experimental design is shown in Figure 1. ‘My website’ denotes the tested website, on which the user adds items to the cart (‘User’s selections’). They are analyzed (‘Summary of selections’) together with data from eye tracking and the supplementary survey to propose the final conclusions on user behavior and the recommendations for e-commerce website design in terms of product highlighting.

### 3.2. Experimental Sessions and Data Collection

The task for each of the participants in the experimental sessions was the same: to select a set of furniture. For this purpose, each of them had to choose one of ten products in a category. This activity was repeated for six different furniture categories: chairs, tables, sofas, armchairs, beds, and wardrobes.

The research was performed on a sample of *n* = 30 participants. All participants were IT students between the ages of 19 and 25 (mean: 22), with 26 male (87%) and 4 female (13%). Most of them shopped online at least once a month (67%), and none had never shopped online before. None of the respondents declared that they had a problem with navigating online stores. It can be stated that the study participants represented rather advanced computer users.

Each session was recorded using research-grade eye movement sensing equipment, Tobii Pro X3 120 Hz gaze tracker and Tobii Pro Lab software. In addition to that, users’ selections (tracking cart’s contents) were saved in a NoSQL document database—Firestore from the Firebase tool. All studies were conducted anonymously, and to identify data from an individual user random identifiers were used.

At the beginning of the experimental session, each participant was asked to sit comfortably at the test stand, which consisted of the gaze tracker, an AOC 24” LCD (I2490VXQ) monitor with a resolution of 1920 × 1080 pixels, and a mouse. Prior to eye tracker calibration, the participants were informed about how eye tracking sensors worked and were asked not to change their sitting position during the test. In the case of obtaining unsatisfactory results, the calibration was repeated. Then, the participants familiarized themselves with the instructions on how to complete the task.

When a participant was ready to begin the test, they pressed the button “Start Test”, which started the whole process. The user could choose any product in a category and after 15 s was redirected to the next category. After selecting a product in all six categories, a screen summarizing the user choices was displayed.

Next, the user was transferred to a survey on the Google Forms platform. The questionnaire contained questions about basic demographics (age, sex), online shopping frequency, ease of Internet use, and 7 proper questions about the impact of the highlights on their decisions during the study. The answers were quantified on the 5-point Likert scale.

The entire examination in the experiment lasted about 10 min per person.

### 3.3. Experimental Website

**Figure 2:**
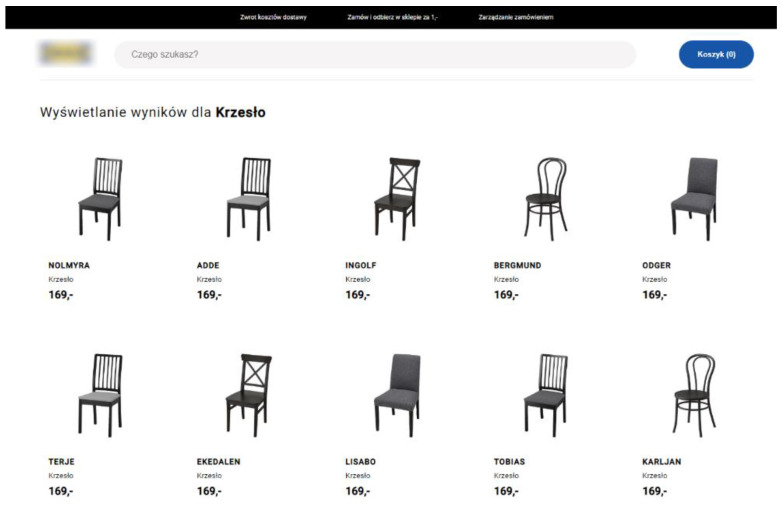
Test store appearance—category page. Source: own elaboration.

In our experiment, the website for research purposes was made in the style of a popular furniture online store, IKEA. At the top of the page (Figure 2) there were such elements as a logo, search box, and a cart with information about the number of products in it. However, they had no full functionalities, and were only complementing the main-content interface of the website. The main content was information about a given category and related products. They were arranged in a grid of 2 rows with 5 products each.

As the experimental stimuli, we wanted to choose products that would not be very prone to user subjectivity. Unfortunately, there are no products that can completely eliminate this factor. We also wanted to choose products with which the users had contact on a daily basis, so that they would be easily identifiable by each participant. Furniture meets these criteria and, therefore, we decided to focus on it in this study.

It is important to note that when selecting the products assortment for the shop, we tried to include pieces of furniture that were as similar as possible to each other. This was to minimize the differences resulting from the aforementioned subjective style perceptions of users. In addition, the same product in each category was listed twice. The website was adapted so that the test person had a view of all products without the need to scroll the page, since scrolling could introduce an additional distraction. This allowed the user to focus less on the browsing action itself and more on the products and highlights. Each participant received the same page—including the order of products in each category. The user had 15 s to choose one item in a particular category. During that time the user was able to change their decision. After the time elapsed, they were transferred to the next category. This process was repeated until all required products were added to the shopping cart. Additionally, information about the remaining time was displayed in the lower right corner.

We have tested the following types of highlights: colored background, shadow, animation, and border. Each highlight type had been examined at 3 levels of intensity to help attract user attention in many ways. For each user, all the highlights were assigned to the same products and were in different places of the grid for each category.

**Figure 3:**
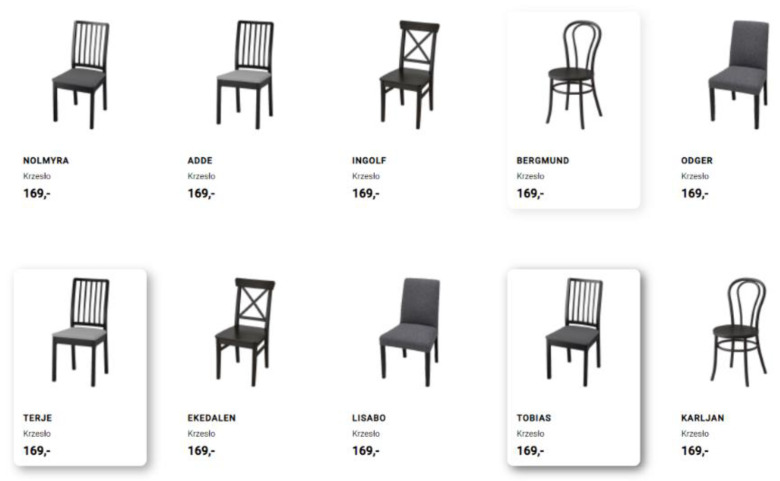
Sample products with different intensities of shadow. Source: own elaboration.

**Figure 4:**
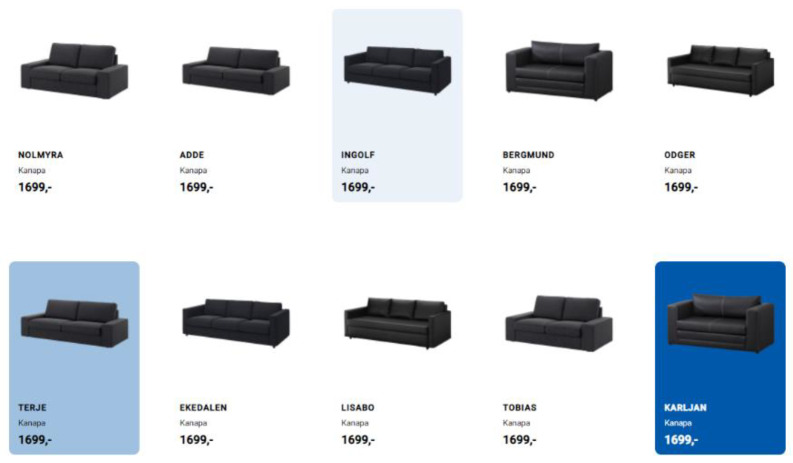
Sample products with different intensities of colored background. Source: own elaboration.

There were 3 highlighted products in each of the categories. Each of these products used a different intensity on a scale of 1–3. The shadows cast by the tile with the product (Figure 3) and the colored backgrounds (Figure 4) were tested in two product categories each. For the shadows, the intensities available included: light, medium, and strong shadow. For the backgrounds, the intensities tested included: tint blue, light blue, and dark blue.

Various types of borders and animations in one product category were also examined. Borders had three intensities: thin, thick dotted, and thick solid. The differentiation of animation intensity was obtained by using 3 levels of movement frequency: slow, medium, and fast.

The website was created so that the user could view it completely without scrolling. This allowed the user to focus less on the action itself and more on the highlights.

### 3.4. Data Analysis

As mentioned earlier, data analyzed in the study came from three sources. The first was the fixation time results collected by the Tobii Pro Lab software tool from gaze tracking. The system allowed for the export of data in legible spreadsheet tables, which facilitated an efficient analysis. Another source of data was the information about the products selected by a user saved in the database using the Firebase tool. The data was exported to the JSON format. To extract the necessary information from it, a simple script was written in JavaScript, which counted the selected products for individual highlights. The last source of data for our analyses were the completed surveys on Google Forms, where the user responses were analyzed in the web application, as well as MS Excel.

## 4. Results

In this section we concentrate on the overall results of product selections, and then we delve into each of the four methods of product highlighting, followed by a summary of the results and a discussion.

### 4.1. Overall Results of Product Selections

Due to the predetermined time slot allowed per product category, the task always took 1.5 min. In total, 30 people chose 175 products, which meant that there were 5 times in which there was a situation where the user did not manage to finalize their choice in a category within 15 s. The eye tracking data in the study was limited to 14 s per category after a subtraction of 0.5 s at the beginning and end of each category, for the duration of page transition. Out of all selections, 53 were featured products, which constituted over 30% of all the choices.

**Figure 5:**
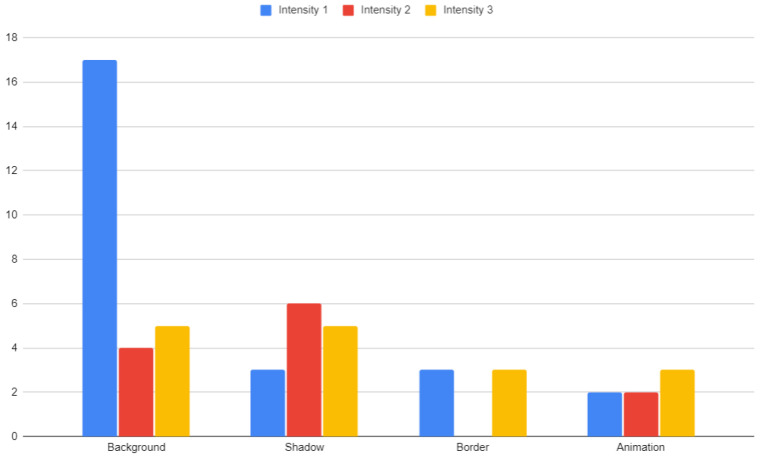
The number of user selections for featured products. Source: own elaboration.

The distribution of user selections regarding highlight types and the level of their intensity are depicted in Figure 5. For each of the categories, the user was shown a set of 10 products, in which 3 of them were highlighted with 3 levels of intensity. Background and shadow highlights were used in two categories, and borders and animations in one.

In the supplementary survey, 30% of study participants claimed that their choices were rather influenced by the highlights (no strong opinions noted), while 57% did not feel their selections were influenced by the highlights (7% felt strongly about it). It is interesting that the respondents in the survey reacted to any highlight (regardless of its type) similarly as to product animation, which was confirmed by a high Cramér’s V correlation of 0.65.

### 4.2. Background Highlight

One type and intensity of highlight in our study showed a significant advantage—the background highlight with the weakest intensity, which resulted in over 4 times more selections than the medium intensity background, and over 3 times more than the high intensity background. If we consider the selections of products with background highlights of all 3 intensities, they account for 26 out of 53 choices, which is almost 50% of all selected highlighted items. However, respondents did not clearly confirm that in their questionnaires, with almost the same percentage claiming that this type of highlight did influence their decisions (45% of responses) as those claiming it did not make any effect on their choices (48% of responses).

The categories with background highlights achieved an average of 1.33 s of fixation time per element, while non-highlighted ones obtained 0.7 s per element. Detailed values for individual products are presented in the tables below (Table 1 and Table 2). The individual numbers in P*i* (P1…P10) indicate the product order, starting from the top left corner (P1…P5 in the first row, and P6…P10 in the second row of the product grid).

**Figure 6:**
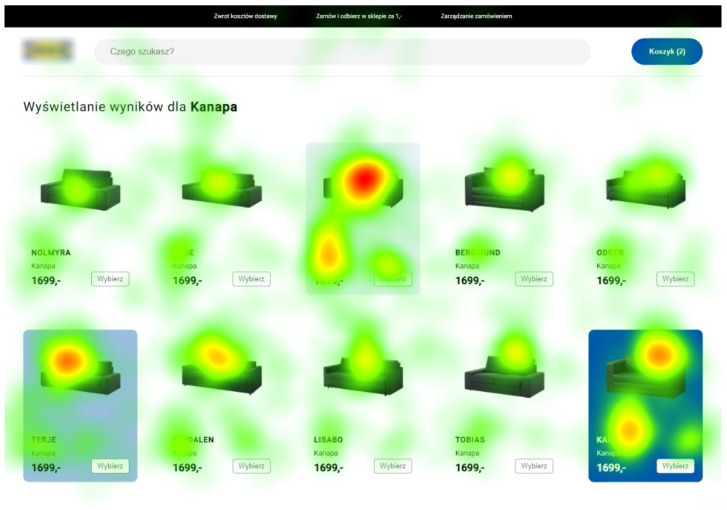
Heatmap for the first product category with background highlights. Source: own elaboration.

**Figure 7:**
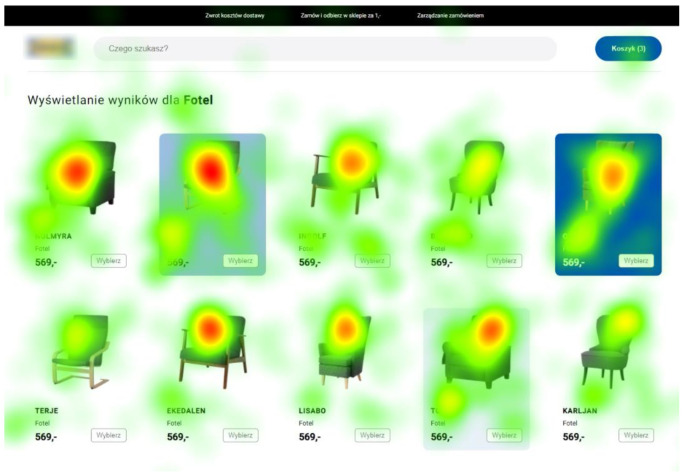
Heatmap for the second product category with background highlights. Source: own elaboration.

What is important is that our eye tracking study confirmed a higher level of interest in highlighted items, regardless of the location of the product. In the pictures below, for example, we can see a greater fixation time for a low-intensity background, both in the P3 position (Figure 6) and P9 (Figure 7).

For both product categories with background highlights, the average fixation time was twice as high as for non-highlighted products. Although the highlighted products constituted 30% of all products, the total fixation time on them was almost 45% of the fixation time on all products. In terms of fixation times, the background with the lowest intensity obtained the best results—for both categories it obtained on average 3.29 s per person, while the medium intensity was 2.22 s, and the highest intensity—2.45 s. These results were concordant with the users’ final product selections.

### 4.3. Shadow Highlight

The weakest shadow intensity did not seem to attract special attention from users (P4 in Table 3 and P4 in Table 4) in terms of fixation altogether, and products with shadow highlight at grades 2 and 3 did not achieve better results (P6 and P9 in Table 3, and P1 and P7 in Table 4, respectively). However, the products highlighted with stronger intensities (level 2 and 3) were more frequently selected by users—6 times in the case of intensity 2 and 5 times in the case of intensity 3, while only 3 times for intensity 1. In the survey, 8% the of answers represented a strong and 57% a weak disagreement with the influence of shadows on product selections, while 5% and 23%—respectively, strong and weak agreement.

### 4.4. Borders

Although the highlights in the form of borders (frames) were chosen the least frequently, the fixation results for them stand out. Frames with intensity level 2 obtained the highest fixation time of all intensities (P2 in Table 5)—14.47% of fixation time of all products in the category. The other intensities did not fare much worse. The weakest and the strongest intensity, for products P5 and P9, respectively, attracted 11.81% and 13.96% of fixation time of all products in the category. Nevertheless, it did not affect the product choices of users, who selected products with a frame only 4 times in total. In the supplementary survey, 23% the of participants presented strong and 33% weak disagreement with the influence of border on their product selections, while 10% and 27%—respectively, strong and weak agreement.

### 4.5. Animations

For animations with the highest intensity, the highest fixation on a single product in the entire study was recorded (P4 in Table 6), which accounted for almost 25% of the total fixation time for all products in this category. Despite such high fixation times, the product with the highest animation intensity was selected only 3 times. In the supplementary survey, almost the same number of respondents disagreed that they were influenced by animations (50%, out of which 13% strongly and 37% weakly) and agreed with that influence (43%, out of which 23% strongly and 20% weakly).

### 4.6. Summary of Results and Discussion

Based on the analysis of the presented results, we can confirm that the impact of too intensive highlighting for recommended items in e-commerce may be negative in terms of user attention. This was visible in the case of the most intense animation. Despite the record time of fixation, it was not often chosen by users. It being too strong invasively may have resulted in its avoidance by the examined person. This is in line with other recent studies [13,14,18,24]—a tension exists between generating interest and triggering annoyance in users, and as such, designing an effective advertising attempt is not an easy task [25].

Moreover, the example with the animation demonstrated that the fixation time was not always related to the user’s product selection. Due to the fact that the products presented were almost identical, it can be concluded that it was the movement of the product that had a negative impact on its perception in our study. This is concordant with a recent study by Oliveira et al. [26] and Coskun et al. [27]—the success of animation in attention-guiding may not have a positive impact on the ultimate goal of a website.

Alternatively, if the highlights are adjusted in the right way, with the right intensity, they could improve the fixation time and encourage the user to buy a product. This was felt in our study in the case of background highlights. The average user fixation time and the number of total selections for products highlighted in this way was higher than for non-highlighted ones. The weakest intensity obtained the best results for this distinction, both in the case of fixation and the number of choices made by the participants.

Additionally, it should be noted that a well-adjusted highlight brought the expected results, regardless of its position on the page. While a user almost always pays attention to the center of the screen, an effective highlight applied even to the right edge would not be omitted, as shown in the case of background highlight.

Results of the follow-up survey showed that users may often be unaware of the influence of marketing attempts in the form of highlights on their product selections while shopping online. This was in contrast with a survey by Bongard-Blanchy et al. [28], where the respondents were found to be generally aware of the influence of manipulative website designs. An open question is whether such awareness may equip users with the ability to oppose that influence.

## 5. Conclusions and Future Work

In today’s e-commerce sites and high competition, user attention is extremely important, and e-commerce websites need to develop their quality [29].

The main conclusions of our experiment are summarized below:

- Attracting attention in the wrong way can have negative results on user perception. Too much highlighting intensity can be irritating and discouraging for the recipient. Our study confirms that, for example in the product category with animations, where an intensely animated product achieved the best fixation time among all the elements, but did not bring the expected add-to-cart results—probably due to its very high invasiveness.
- An appropriate selection of highlights and intensities can obtain better fixation results, while at the same time prompting the user to select a product, even when the product is in less attractive, less central website areas that are often avoided when viewed without any highlights present. Most user selections concerned the lowest intensity background highlight.
- The fixation time was also influenced by the location of the product in the grid, however, proper selection of the highlights increased the fixation time regardless of the position, which was shown in our study in the case of background highlights.

When analyzing all the above results, we are aware that the limitation of the study was the relatively small group of respondents, so our results may differ from those obtained on another research sample. Moreover, it should be remembered that each test person is different, and different people may have different visual preferences [30], so human-computer interaction studies such as ours are often just an attempt at generalization [31]. It should also be remembered that apart from the effect of highlights themselves, different users may perceive the same products differently. Although the products in the study were very similar, they were not identical and minimal differences may still have affected the final decisions. Finally, in the study all product prices were the same. In reality, price could be a deciding factor in the final choice.

If invasive attempts to attract eyesight in e-commerce may have negative effects, how can one properly choose a good product distinction? Certainly, a study such as this can precheck its effectiveness, while full eye tracking research would cost a great deal of money. It seems a good idea to choose lower intensities while highlighting products. In this case, if the highlighted products did not obtain better results than the rest of the products in our study, at least they did not seem to suffer from negative effects of attempts to attract user’s attention. With such an approach, without further potential costly site testing, we should not lose anything, and potentially we can gain something.

Our next step in the research will be to study the influence of more types of animations and background highlights in different colors. The methods of highlighting we examined, as well as other types of product distinction, should be considered and adapted to the context of a particular website. It is recommended that before implementing product highlighting on a website, they should be properly tested with different levels of intensity to avoid effects opposite to those intended.

## Figures and Tables

**Table 1:** Fixation times in the first category with background highlights. Source: own elaboration.

	P1	P2	P3	P4	P5	P6	P7	P8	P9	P10
Intensity	-	-	1	-	-	2	-	-	-	3
Average (s)	0.49	0.56	2.08	0.53	0.60	0.93	1.06	0.71	0.47	1.48
Share of Total Time (%)	5.44	6.30	23.31	6.00	6.75	10.44	11.90	7.95	5.30	16.62

**Table 2:** Fixation times in the second category with background highlights. Source: own elaboration.

	P1	P2	P3	P4	P5	P6	P7	P8	P9	P10
Intensity	-	2	-	-	3	-	-	-	1	-
Average (s)	1.10	1.29	1.13	0.82	0.97	0.34	0.75	0.66	1.21	0.57
Share of Total Time (%)	12.48	14.60	12.80	9.26	10.95	3.86	8.43	7.47	13.66	6.49

**Table 3:** Fixation times in the first product category with shadow highlights. Source: own elaboration.

	P1	P2	P3	P4	P5	P6	P7	P8	P9	P10
Intensity	-	-	-	1	-	2	-	-	3	-
Average (s)	0.46	0.67	1.19	0.83	0.80	0.65	0.66	1.59	0.89	0.33
Share of Total Time (%)	5.75	8.31	14.68	10.29	9.91	8.07	8.18	19.62	11.05	4.14

**Table 4:** Fixation times in the second product category with shadow highlights. Source: own elaboration.

	P1	P2	P3	P4	P5	P6	P7	P8	P9	P10
Intensity	2	-	-	1	-	-	3	-	-	-
Average (s)	1.06	0.62	1.02	0.97	0.84	0.41	0.77	1.01	1.36	0.46
Share of Total Time (%)	12.47	7.28	11.98	11.37	9.85	4.82	9.11	11.85	15.93	5.35

**Table 5:** Fixation times in the product category with borders. Source: own elaboration.

	P1	P2	P3	P4	P5	P6	P7	P8	P9	P10
Intensity	-	2	-	-	1	-	-	-	3	-
Average (s)	0.58	1.22	1.43	0.69	1.00	0.41	0.84	0.76	1.18	0.34
Share of Total Time (%)	6.84	14.47	16.95	8.18	11.81	4.81	9.92	9.02	13.96	4.04

**Table 6:** Fixation times in the product category with animations. Source: own elaboration.

	P1	P2	P3	P4	P5	P6	P7	P8	P9	P10
Intensity	1	-	-	3	-	-	2	-	-	-
Average (s)	0.67	0.76	1.51	2.32	0.50	0.19	1.21	0.79	0.97	0.47
Share of Total Time (%)	7.14	8.06	16.13	24.71	5.32	2.00	12.86	8.38	10.39	5.02

## Data Availability

Some data presented in the study are available from the corresponding author, upon request, data sharing being limited due to personal information content, and the code used during the study being proprietary.
